# Editorial: Impact of neurostimulation and psychotherapy on mental health

**DOI:** 10.3389/fpsyg.2022.992634

**Published:** 2022-08-09

**Authors:** Lence Miloseva, Kneginja Richter

**Affiliations:** ^1^Faculty of Medical Sciences, Goce Delcev University, Stip, North Macedonia; ^2^Department of Psychiatry and Psychology, Neuromedica Hospital, Skopje, North Macedonia; ^3^University Clinic for Psychiatry and Psychotherapy, Paracelsus Medical University Nuremburg, Nuremburg, Germany; ^4^Faculty for Social Sciences, Technical University for Applied Sciences Nuremberg, Nuremburg, Germany

**Keywords:** neurostimulation, psychotherapy, CBT, rTMS, neurofeedback, mental health

Brain stimulation therapies as biologically-oriented treatment methods can play an important role in treating certain psychological disturbances and psychiatric diseases. Step by step they successfully have been implemented in the field of mental health together with the different psychotherapy methods. While these types of therapies are less frequently used than medication and psychotherapies, they hold promise for treating certain mental disorders that do not respond to other treatments. These have included depression, posttraumatic stress disorder, anxiety disorder, Alzheimer's disease, and dementia. Non-invasive brain stimulation (NIBS) has been proposed as an intervention strategy for mental disorders. NIBS has immediate effects on neural excitability but also after effects (Klomjai et al., 2015) which makes it a potentially suitable therapeutic tool for mental disorders. The most frequently utilized neurostimulation methods are repetitive transcranial magnetic stimulation (rTMS) and electroconvulsive therapy (ECT). Last years, many new methods are emerging in the field of mental health, which mainly include transcranial direct current stimulation (tDCS) and neurofeedback.

Psychotherapy, especially Cognitive Behavioral Therapy (CBT) oriented modalities is a psychologically-oriented treatment method which can lead to behavior change, changes in cognition, emotions and significant improvements in psychological symptoms and mental health. Contemporary Psychotherapy modalities include Cognitive Behavioral Therapy (CBT), Rational Emotional Behavioral Therapy (REBT), Acceptance Commitment Therapy (ACT), Mindfulness and Schema therapy, among others. While neurostimulation and psychotherapy use different mechanisms, both can influence and change human psychological states and mental health. That is the main reason why this Research Topic attracts scientific attention for future discussion.

This Research Topic offers several investigations into the empirical data and related papers comparing the effectivity of new biologically-driven treatment methods based on neurostimulation and the psychologically-driven treatment methods based on psychotherapy.

The first article of this Research Topic (Teoh et al.) is a systematic review and meta-analysis aimed to critically evaluate and determine the effectiveness and safety of Mindfulness-based Interventions in alleviating the feeling of loneliness. Despite several methodological limitations, the review found significant improvement in loneliness when mindfulness intervention with an average length of 8-week duration was introduced to the population with generally no mental health issues. Given the current rise in loneliness levels, clinicians and the public can consider applying mindfulness intervention to alleviate loneliness when there is no existing mental health condition.

There is a growing interest in combining TMS with other concurrent psychotherapeutic interventions to optimize treatment outcomes. A very interesting article on this Research Topic (Cavallero et al.) is a pilot study that underscored feasibility issues surrounding concurrent TMS administration and mindfulness-based practice in the form of listening to audio-guided meditation exercises. They concluded that It is possible that learning and practicing Mindfulness Cognitive Behavioral Therapy (MCBT) immediately before or immediately after TMS treatment sessions would have worked better for achieving the goals they identified. The results of their study highlight numerous feasibility issues with MBCT *via* guided audiotapes during TMS treatment. Future work should draw on these shortcomings to evaluate the appropriateness of MBCT for depressed patients undergoing neuromodulation.

The part of this Research Topic is also a pilot study (Carstens et al.) aimed to investigate the effects of a half-open continuous group cognitive behavioral therapy (CBT) with a cognitive behavioral analysis system of psychotherapy elements as a follow-up treatment for all ECT patients, regardless of response status after ECT, on reducing depressive symptoms and promoting psychosocial functioning. During group CBT, Post-ECT symptom reduction was not only maintained but there was a tendency toward a further decrease in depression severity. This reduction could be sustained 6 months after the end of the group, regardless of response status after ECT treatment. Aspects of quality of life and emotion regulation strategies improved during group CBT, and these improvements were maintained 6 months after the end of the group. Their approach might not only help to further reduce depressive symptoms and prevent relapse but also promote long-term psychosocial functioning by improving emotion regulation strategies and psychological quality of life. A study conducted by Lyby et al. aimed to compare two multidisciplinary occupational rehabilitation programs based on multimodal cognitive behavioral therapy on self-rated health and work ability. The results revealed that both groups improved on the selected outcomes. Within-group contrasts and effect sizes showed that the inpatient group showed larger effect sizes at the end of rehabilitation and 12 months post-intervention for work-related outcomes than the outpatient group.

The next two articles are focused mostly on the impact of rTMS on mental health. The article of Grycuk et al. aimed to explore experiences and perceptions of transcranial direct current stimulation (tDCS) among participants diagnosed with schizophrenia. Five themes were identified: (a) motivation for study enrolment; (b) concerns about tDCS; (c) factors reducing the fear of tDCS; (d) experience of tDCS; (c) perceived effects of tDCS. After the first session, participants no longer felt anxious about the remaining ones. Strategies to improve treatment experience and study recruitment have been identified. Future perspectives from a case report on Transcranial Magnetic Stimulation Cognitive Behavioral Therapy, and Psychopharmacological Treatment for Post-traumatic Stress Disorder were presented in the article of Seybert et al. The last article on this Research Topic is focused on *Risk assessment of electroconvulsive therapy in clinical routine: A 3-year analysis of life-threatening events in more than 3,000 treatment sessions* (Hajak et al.). The beneficial risk profile of ECT performed in the standard care of psychiatric hospitals suggests a more generous indication of this treatment method. They recommend that ECT facilities collect their own safety data to allow a reliable judgment of their institutional ECT risk profile.

This Research Topic gathers different contributions highlighting novel impacts of Neurostimulation and Psychotherapy on Mental Health (Richter et al., 2017). This remains to be seen, but the seven articles that comprise this Research Topic offer valid scientific insight into how Neurostimulation and Psychotherapy could combine to benefit the field of mental health. These approaches allow us to shed light on the mechanisms of impact and improvement of contemporary treatments of mental disorders.

## Author contributions

LM wrote the first draft. KR provided critical comments and Editorial suggestions for revisions. All authors agreed on the submitted version.

## Conflict of interest

The authors declare that the research was conducted in the absence of any commercial or financial relationships that could be construed as a potential conflict of interest.

## Publisher's note

All claims expressed in this article are solely those of the authors and do not necessarily represent those of their affiliated organizations, or those of the publisher, the editors and the reviewers. Any product that may be evaluated in this article, or claim that may be made by its manufacturer, is not guaranteed or endorsed by the publisher.
